# Level of physical activity and associated factors during pregnancy among women who gave birth in Public Zonal Hospitals of Tigray

**DOI:** 10.1186/s13104-019-4496-5

**Published:** 2019-07-23

**Authors:** Dawit Gebregziabher, Haftu Berhe, Mekuria Kassa, Eskedar Berhanie

**Affiliations:** 1 Lecturer at Nursing School, College of Health Science, Aksum University, Aksum, Ethiopia; 2 School of Nursing, College of Health Science, Mekelle University, Mekelle, Ethiopia

**Keywords:** Physical activity, Exercise

## Abstract

**Objectives:**

Physical activity in the general population is considered too low, and this is true for pregnant women. Moderate physical activity during pregnancy have many benefits for the mother and the developing baby. This study was aimed to assess the level of physical activity during pregnancy and associated factors in public zonal hospitals of Tigray, Ethiopia. A hospital based cross-sectional study was used and 458 study participants was selected using multistage sampling technique. The data were collected using standardized pregnancy physical activity questionnaire.

**Result:**

Out of 442 women who participated in this study, only 21.9% were physically inactive. Parity [AOR = 7.68; 95% CI (3.193, 18.459)], maternal occupation [AOR = .015; 95% CI (.003, .083)], history of miscarriage [AOR = 8.045; 95% CI (3.325, 19.465)], maternal age AOR = 4.67; 95% CI (1.431, 15.254)], were the variables that showed statistical association with level of physical activity during pregnancy. Level of physical activity during pregnancy was generally high. Thus, it would be optimal if health professionals can take a more active role in promoting physical activity during pregnancy.

**Electronic supplementary material:**

The online version of this article (10.1186/s13104-019-4496-5) contains supplementary material, which is available to authorized users.

## Introduction

Physical activity is an important component of a healthy pregnancy, for both the mother and her child. Physical activity has been accepted internationally as an important factor for the protection and improvement of health in pregnant women as well as in the general population. Being physically active is recognized as an essential part of a healthy pregnancy and is associated with improved cardiovascular function and physical fittness and reduced risk of adverse maternal outcomes including gestational diabetes, preeclampsia and preterm birth [1, 2].

According to the World Health Organization recommendations, adults aged 18 to 64 years should have at least 150 min of moderate-intensity, or 75 min of vigorous-intensity aerobic physical activity throughout the week, or an equivalent combination of both [3]. The Centers for disease control and prevention (CDC), American college of sports medicine (ACSM) adopted this recommendation for pregnant women and advised engagement in 30 min of moderate exercise per day on most days of the week, equivalent to 7.5 MET-h/week [4].

Globally, around 23% of adults aged 18 and over were not active enough in 2010 (men 20% and women 27%). Only 10.2% of women fulfilled the recommendations in Brazil, 17.4% in United States, 20.3% in Spain, 47% in France, and 48% in United Kingdom [5–10].

Proper and adequate physical activity during pregnancy has a major impact on mother’s health and fetal growth. The earliest recommendations for PA during pregnancy largely reflected the cultural and social norms of the times, rather than scientific evaluation [11–15]. Even though physical activity during pregnancy is beneficial in the absence of medical or obstetric complications, most of the pregnant women’s may not perceive it safe appropriate and feasible. This is mainly due to lack of information on physical activity during pregnancy, safety issue and cultural norms [16–18].

The finding of this study would, be potentially useful in generating previously unavailable data on the level of physical activity during pregnancy, therefore policy makers and NGOs may use this for future planning and public health interventions of appropriate strategies to prevent pregnancy comorbidities and promote maternal and fetal health.

## Main text

### Materials and methods

This study was conducted in three randomly selected zonal public hospitals of Tigray Regional state of Northern Ethiopia from Oct. 2016 up to May 2017. Tigray is one of the nine ethnic regions in Ethiopia. Tigray is divided into 47 districts (woredas) which are grouped into six administrative zones like Eastern zone, Central Zone, North Western Zone, Southern Zone, Western Zone, Mekelle (special zone). The study design was hospital based cross-sectional study design [19].

The study population includes sampled women who gave birth in selected Zonal Hospitals of Tigray. A total of 458 samples was employed among the three selected zonal hospitals using systematic random sampling.

Data was collected using standardized pregnancy physical activity questionnaire (See Additional file 1) [20]. The intensity of each activity was estimated using 2011 compendium-based metabolic equivalent (MET) values. The intensity of activities was multiplied by the duration of time spent on each activity to get average weekly energy expenditure. Metabolic equivalent task was (1 MET = 1 kcal/kg × hour) values was used to classify each activity by intensity [21]. Data was entered using Epi info version 7, cleaned and analyzed using Statistical package for social sciences version 22.0 software. Descriptive statistics was used to characterize the sample. Logistic regression was used to assess statistical association and significance of statistical association was assured or tested using 95% confidence interval and P value (< .05). Ethical clearance was secured from the Mekelle University, college of health science institutional review board. Oral and written consent was obtained from each participant. Information was recorded anonymously and confidentiality and beneficence were assured throughout the study period.

### Result

#### Maternal socio demographic characteristics

A total of 458 women aged 18–38 participated in the study giving over all response rates of 96.5%. The reasons for non-response were: incomplete physical activity data due to missing activity recordings (n = 11), refusal to participate (n = 5). The median (± IQ range) of age of mothers were 26 ± 08 years range from 18–38 years. Two hundred and seventy-two (60.9%) of the respondents were in the age range of 25–40 years (Table 1).

**Table 1:** Distribution of socio-demographic characteristics of women by the level of physical activity during pregnancy in selected zonal hospitals of Tigray, Ethiopia (n = 442), 2017

Variables	Category	Total (%)	Level of PA	
			Active	Inactive
			n = 345 (%)	n = 97 (%)
Age (years)				
	< 19	36 (8.1)	22 (6.4)	14 (14.4)
	20–24	134 (31.0)	107 (31.0)	27 (27.8)
	25–29	154 (34.8)	124 (35.9)	30 (30.9)
	30–34	81 (18.3)	69 (20)	12 (12.4)
	35–40	137 (8.4)	23 (6.7)	14 (14.4)
Marital status				
	Married	384 (86.9)	310 (89.9)	74 (76.3)
	Never married	30 (6.8)	15 (4.3)	15 (15.5)
	Divorced/widowed	28 (6.3)	20 (5.8)	8 (8.2)
Education level				
	Illiterate	68 (15.4)	26 (7.5)	42 (43.3)
	Elementary school	140 (31.7)	130 (37.7)	10 (10.3)
	High school	142 (32.1)	117 (33.9)	25 (25.8)
	College/university	92 (20.8)	72 (20.9)	20 (20.6)
Occupation				
	House wife	150 (33.9)	95 (27.5)	55 (56.7)
	Govt. employee	90 (20.4)	72 (20.9)	18 (18.6)
	Private employee	63 (14.3)	55 (15.9)	08 (8.2)
	Farmer	95 (21.5)	91 (26.4)	04 (4.1)
	Trader	22 (5.0)	18 (5.2)	04 (4.1)
	Student	22 (5.0)	14 (4.1)	8 (8.2)
Income^a^ (n = 394)				
	< 1000 birr	104 (23.5)	89 (27.1)	15 (18.3)
	1001–1500	44 (10)	35 (10.7)	9 (11)
	1501–2000	82 (18.6)	70 (21.3)	12 (14.6)
	2001–3000	108 (24.4)	81 (24.7)	27 (32.9)
	3001–above	72 (16.3)	53 (16.2)	19 (23.2)

^a^Cell counts may not add up to total number of participants due to missing values

#### Maternal obstetrics characteristics

Four hundred thirty (97.3%) of women had received ANC service at least once during the index of pregnancy. Only 90 (20.4%) of the women reported previous history of miscarriage. The median (± IQ range) of parity was 1 ± 2 ranges from 1 to 8 live births and more than half 247 (55.9) were multiparous and 341 (77.1%) of the pregnancy were planned (Table 2).

**Table 2:** Distribution of obstetrics characteristics of women by their level of physical activity during pregnancy in selected zonal hospitals of Tigray, Ethiopia, 2017 (n = 442)

Variables	Category	Frequency (%)	Level of PA	
			Active	Inactive
			n = 345 (%)	n = 97 (%)
ANC follow up				
	No	12 (2.7)	10 (2.9)	2 (2.1)
	Yes	430 (97.3)	335 (97.1)	95 (97.9)
ANC type^a^ (n = 430)				
	Public ANC	286 (66.5)	212 (61.4)	74 (76.3)
	Private ANC	144 (33.5)	123 (35.7)	21 (21.6
Pregnancy intention				
	Planned	341 (77.1)	290 (84.1)	51 (52.6)
	Unplanned	101 (22.9)	55 (15.9)	46 (47.4)
Parity				
	1 primipara	195 (44.1)	125 (36.2))	70 (72.2)
	≥ 2 multipara	247 (55.9)	220 (63.8)	27 (27.8)
History of miscarriage				
	No	352 (79.6)	288 (83.5)	64 (66.0)
	Yes	90 (20.4)	57 (16.5)	33 (34)

^a^Cell counts may not add up to total number of participants due to missing values

#### Physical activity characteristics of women during pregnancy

The median total weekly energy expenditure (MET-h/week) of physical activity during the entire trimester of pregnancy was 141.23 MET-h/weeks [(Mean ± SD) 140 ± 62.46]. More than three fourth 345 (78.1%) of women were doing 30 min or more of moderate-intensity physical activity on most days of the week and the remaining 97 (21.9%) were not doing physical activity as per the recommendation.

#### Factors affecting the level of physical activity during pregnancy

Multivariable logistic regression was done by taking 10 variables in to account simultaneously. After testing for assumptions, logistic regression was conducted using the default method.

The half of variables which showed significant association with level of physical activity during pregnancy in bivariate analysis could not persist as significant in the multivariable analysis. These covariates were; ANC type, marital status, pregnancy intention, perceived benefit of activity and perceived barrier to exercise.

In the multivariable binary logistic regression analysis, only five variables had shown overall Significant effect on level of physical activity during pregnancy at 5% level of significance.

Based on this, parity, maternal previous history of miscarriage, Education level of mothers, maternal age and occupation were variables which significantly associated with physical activity level (Table 3).

**Table 3:** Bivariate and multivariable logistic regression analysis result of level of physical activity and associated factors during pregnancy among women who delivered in selected zonal Hospitals of Tigray, Ethiopia, 2017 (n** = **442)

Variable	Category	n (%)	Level of PA		COR [95% CI]	AOR [95% CI]
			Active	Inactive		
			n = 345 (%)	n = 97 (%)		
Age (years)						
	< 19 years	36 (8.1)	22 (6.4)	14 (14.4)	2.630 [1.206, 5.737]*	4.673 [1.431, 15.254]**
	20–24	134 (31.0)	107 (31.0)	27 (27.8)	1.043 [.584, 1.864]	1.570 [.656, 3.757]
	25–29	154 (34.8)	124 (35.9)	30 (30.9)	1	
	30–34	81 (18.3)	69 (20)	12 (12.4)	.719 [.346, 1.494]	1.948 [.544, 6.978]
	35–40	137 (8.4)	23 (6.7)	14 (14.4)	2.516 [1.159, 5.460]*	3.348 [.792, 14.149]
Marital status						
	Married	384 (86.9)	310 (89.9)	74 (76.3)	1	1
	Never married	30 (6.8)	15 (4.3)	15 (15.5)	4.189 [1.961, 8.951]*	3.191 [.786, 12.950]
	Divorced/widowed	28 (6.3)	20 (5.8)	8 (8.2)	1.676 [.710, 3.953]	3.778 [.844, 16.902]
Educational level						
	Illiterate	68 (15.4)	26 (7.5)	42 (43.3)	7.560 [3.937, 14.517]*	19. 359 [4.926, 76.077]**
	Elementary school	140 (31.7)	130 (37.7)	10 (10.3)	.360 [.166, .781]*	.260 [.087, .775]**
	High school	142 (32.1)	117 (33.9)	25 (25.8)	1	
	College/university	92 (20.8)	72 (20.9)	20 (20.6)	1.300 [.674, 2.508]	1.699 [.248, 11.662]
Occupation						
	House wife	150 (33.9)	95 (27.5)	55 (56.7)	1	1
	Govt. employee	90 (20.4)	72 (20.9)	18 (18.6)	.432 [.234, .798]*	1.617 [.172, 15.185]
	Private employee	63 (14.3)	55 (15.9)	08 (8.2)	.251 [.111, .566]*	.655 [.162, 2.654]
	Farmer	95 (21.5)	91 (26.4)	04 (4.1)	.076 [.026, .218]*	.015 [.003, .083]**
	Trader	22 (5.0)	18 (5.2)	04 (4.1)	.384 [.124, 1.192]	1.166 [.242, 5.614]
	Student	22 (5.0)	14 (4.1)	8 (8.2)	.987 [.389, 2.502]	.864 [.170, 4.396]
ANC type						
	Public ANC	286 (66.5)	212 (61.4)	74 (76.3)	1	
	Private ANC	144 (33.5)	123 (35.7)	21 (21.6	.489 [.287, .834]*	.934 [.414, 2.107]
	No ANC	12 (2.7)	10 (2.9)	2 (2.1)	.573 [.123, 2.676]	.675 [.077, 5.896]
Pregnancy intention						
	Planned	341 (77.1)	290 (84.1)	51 (52.6)	1	
	Unplanned	101 (22.9)	55 (15.9)	46 (47.4)	4.756 [2.908, 7. 777]*	1.450 [.567, 3. 713]
Parity						
	Primipara	195 (44.1)	125 (36.2)	70 (72.2)	4.563 [2.781, 7.488]*	7.677 [3.193, 18.459]**
	Multipara	247 (55.9)	220 (63.8)	27 (27.8)	1	
History of miscarriage						
	No	352 (79.6)	288 (83.5)	64 (66.0)	1	
	Yes	90 (20.4)	57 (16.5)	33 (34)	2.605 [1.569, 4.326]*	8.045 [3.325, 19.465]**
Perceived benefit (s) of PA						
	Improve general health of mother	120 (66%)	97 (28.1)	23 (23.7)	1	
	Shortened delivery process	19 (10.2%)	17 (4.9)	2 (2.1)	.496 [.107, 2. 301]	.463 [.081, 2.638]
	Minimize excessive GWG	31 (16.7%)	26 (7.5)	5 (5.2)	.811 [.281, 2. 340]	1.077 [.286, 4.052]
	Others^c^	16 (8.6%)	12 (3.5)	4 (4.1)	1.406 [.415, 4. 759]	.839 [.168, 4.194]
	No benefit	256 (57.9)	193 (55.9)	63 (64.9)	1.377 [.805, 2. 353]	1.999 [.599, 6.675]
Perceived barrier to exercise						
	Lack of exercise knowledge	160	128 (37.1)	329 (33)	1	
	Have no exercise habit	51	35 (10.1)	16 (16.5)	1.829 [.902, 3. 708]	.460 [.119, 1.786]
	Fear of miscarriage	33	25 (7.2)	8 (8.2)	1.280 [.528, 3. 103]	.493 [.097, 2. 519]
	Others	32	18 (5.2)	14 (14.4)	3.111 [1.400, 6. 915]*	1.378 [.309, 6. 140]
	No barrier	166	139 (40.3)	27 (27.8)	.777 [.441, 1.368]	.534 [.124, 2. 295]

*AOR* adjusted odds ratio, *COR* crude odds ratio

* P value < .05 in the bivariate analysis

** P value < .05 in the multivariable analysis

### Discussion

This study was aimed to assess the level of physical activity and associated factors during pregnancy. Approximately three-fourths (78.1%) of the women in this study physically active and the remaining 97 (21.9%) [95% CI (.181, .258)] were not doing physical activity during pregnancy as per the recommendation. This is lower than the study findings of Ibadan (Nigeria) [18], Campinas (Brazil) [22], United States [23] and Ireland [24] which was 49.0%, 30.8%, 77.5% and 78.5% respectively. The discrepancies in the level of physical activity between other studies and ours may be due to differences in sample characteristics, measurements used (subjective vs objective) and high altitude. Another possible reason is since more than half of the pregnant mothers are house wife and have low and middle income are less likely to outsource housework and other services. This could have raised the physical activity level of the women.

In addition, women in this study expended less total energy during pregnancy 141.23 MET-h/week (approximately 20.2 MET h/day) than pregnant women in the United States (25.4 MET h/day) [25], Australia (33.26 MET h/day) [26], Hong Kong (25.23 h/day) [27], Taiwan (35.9 MET-h/day) [28] and France (29.0 MET h/day) [9] but it is more or less comparable with the studies conducted in Tianjin, China (18.50 to 21.90 MET h/day) [29] and Japan (19.7 MET-h/day) [30]. This might be due to physical activity measurement methods were developed in economically developed countries with the assumptions about body weight. Maternal body weights are likely to be substantially lower in Ethiopia and so less energy is expended for the same level of physical activity.

In this study women expended highest amount of energy (69.4 MET-h/weeks) on household activities. This is comparable with the study conducted previously in Nigeria [18], Hong Kong [27] and Japan [30] which was 63.4 MET-h/week, 79.0 MET-h/week respectively. In addition the median amount of weekly energy expanded on occupation activity (60 MET-h/week) was significantly lower as compared to studies conducted previously in Taiwan (120 MET-h/week) [28] and but higher as compared to another study conducted in in Hong Kong (33.6 MET-h/week) [27] and Japan (00 MET-h/week) [30]. The observation that 56.7% of the pregnant women seen in this study were housewives, accounted for the low energy expenditure on occupational activity. In addition, most the pregnant women’s usually take maternity leave early which may make them spend more time at home than at work. Another possible reason is that most of the pregnant women may feel more comfortable and safer doing household activities than engaging in occupational or sports activities during pregnancy.

This study also showed that primiparous women were 7.68 times more likely to be inactive as compared to multiparous women. This finding is in agreement with prior studies in Nigeria [18], Brazil [31], Massachusetts [32], Botucatu [6], Iowa State (USA), Halifax (Canada), Boston (USA) [25, 33, 34]. This because mothering higher numbers of children requires higher level of activity.

Education level of mothers also had significant association with women’s level of physical activity during pregnancy. Women’s with no formal education were more likely to be in active. Similar findings were also observed in the earlier studies from Australia [35], Brazil [31], Rio Grande [36], Durham [37], Canada [26, 33]. This may be related to the possibility that women with higher educational levels are better informed or have more access to knowledge about physical activity during pregnancy.

Those Women who were engaged in manual occupation were less likely to be physically inactive during pregnancy. This is in line with those of earlier study in Hamadan (Iran) [38], USA [39]. This might be due to the fact that daily occupational activity in Ethiopia use more energy because of the lack of labor saving devices. Another possible reason is because much of Ethiopia is at high altitude, equivalent activity requires greater effort and more energy than it does at sea level. Therefore, women’s living in Ethiopia show high energy expenditure and this may exceed energy intake significantly.

In addition, those women with history of miscarriage had 8.05 times higher odds of becoming physically inactive during pregnancy as compared to those without history of miscarriage [AOR = 8.045; 95% CI (3.325, 19.465)]. This is consistent with the study conducted in Kwun Tong (Hong Kong), Denmark [27, 40]. This might be due to the reason that fear of harming the baby during physical activity and due to the fact that women with previous history of miscarriage are concerned that regular physical activity during pregnancy may cause another miscarriage, poor fetal growth. In addition, the lower levels of physical activity associated with previous miscarriage may be related to prenatal and neonatal practitioners’ advice for more bed rest for women with a history of pre-eclampsia. Thus, they are more likely to become inactive during pregnancy.

This study also showed that women’s whose age was < 19 years had, 4.7 times higher odds of becoming physically inactive during pregnancy as compared those mothers who were in the age range of 25–29 years [AOR = 4.673; 95% CI (1.431, 15.254)]. The findings of the present study are consistent with those of earlier studies that has reported PA association with age Australia, St. Louis (USA), Halifax (Canada), Chapel Hill (USA) [26, 33, 39, 41].

### Conclusion

In conclusion, this study revealed that only about less than one fourth women were in active and most of the physical activities were done during performance of household activities and occupational activity, but there was very little participation in the exercise/leisure time activity domain. On the other hand, this study has also found that both maternal socio-demographic and obstetric factors had contributed for pregnant women’s level of activity.

## Limitation

Lack of abundant previous similar study in Africa made comparison difficult and Self-reported measure of physical activity may lead to recall bias.

## Additional file

**Additional file 1.** Pregnancy physical activity questionnaire.

## Data Availability

All the necessary data and materials of this study are readily available in the additional material section of the journal of BMC in Research Note.

## Abbreviations

AOR: adjusted odds ratios
ACOG: American college of obstetricians and gynecologists
ACSM: American college of sports medicine
ANC: antenatal care
CDC: centers for disease control and prevention
CI: confidence interval
COR: crude odds ratios
IQ: interquartile
MET: metabolic equivalent task
NGO: Non-Governmental Organization
PA: physical activity
PPAQ: pregnant physical activity questionnaire
SD: standard deviation
