# Supplementary material for: Level of physical activity and associated factors during pregnancy among women who gave birth in Public Zonal Hospitals of Tigray
Source: BMC Res Notes. 2019 Jul 23;12:454. doi: 10.1186/s13104-019-4496-5 (PMC6651980; doi:10.1186/s13104-019-4496-5)
Supplement: Supplementary file 1 — Additional file 1. Pregnancy physical activity questionnaire. [file 13104_2019_4496_MOESM1_ESM.docx]

## Annex III: English version pregnancy physical activity questionnaire

**Mekelle University, College of Health Sciences, School of Nursing**

| **Part I. Socio demographic profile** | | | | | | | |
| --- | --- | --- | --- | --- | --- | --- | --- |
| **Name of Hospital** [__________________] | | | **Questionnaire Code**  [_______] | | | | |
| **QNO** | | Question Response | | | | | |
|  | | How old are you | _________[ years old ] | | | | |
|  | | What is your Pre-Gestational Weight? | [ kg ] | | | | |
|  | | What is your relationship status?: [ 1] Single [ 2] Married [ 3] Widow [ 4 ] Divorced | | | | | |
|  | | If Married Do you live with your partner? [ 1 ] Yes [ 2 ] No | | | | | |
|  | | What is your highest level of education completed? | | | | | |
|  | | Parity [ 1 ] No [ 2 ] First [ 3 ]. Second [ 4 ] Third *Other……* | | | | | |
|  | | Occupation of mother  [ 1 ] Farmer [ 2 ] Worker [ 3 ] Student [ 4 ] Un employed  [ 5 ] Trader [ 6 ] Government staff [ 7 ] House wife [ 8 ] Others (*please specify*) | | | | | |
|  | | Monthly income ________ in Ethiopian Birr | | | | | |
|  | | Is your pregnancy planned? [ 1 ] Yes [ 2 ] No | | | | | |
|  | | ANC type [ 1 ] Private [ 2 ] public | | | | | |
|  | | Previous history of Abortion [ 1 ] Yes [ 2 ] No | | | | | |
| **S.N** | | **Part II. Questions about Knowledge and Attitude of mothers with regard to PA during pregnancy** | | | | | |
|  | | **Knowledge of pregnant mothers on physical activity during pregnancy** | | | | | |
| 113 | | Have you ever heard of a physical activity during pregnancy? [ 1] Yes [ 2 ] No (if No let me skip to Q 115) | | | | | |
| 114 | | From where did you hear about physical activity during pregnancy  [ 1] Health worker/Facility [ 2] Mass media (radio, TV) [ 3] Friends (relatives) [ 4] Neighbors [ 5] Others(Specify) | | | | | |
| 115 | | Why physical exercise during pregnancy is necessary? (Specify) | | | | | |
|  | | **Attitude of pregnant mothers on physical activity during pregnancy** | | | | | |
| 116 | | Does physical activity have a positive role in your pregnancy [ 1 ] Yes [ 2 ] No [ 3] I don’t know | | | | | |
| 117 | | Do you think physical activity can reduce pregnancy related complications?  [ 1 ] Yes [ 2 ] No [ 3] I don’t know | | | | | |
| 118 | | Do you recommend physical activity during pregnancy? [ 1 ] Yes [ 2 ] No [ 3] I don’t know | | | | | |
| **Part II Questions about Daily physical activities** | | | | | | | |
| **S.N** | **1. Household/Caregiving activities** | | | | | | |
| 120 | At any time during this pregnancy did you Do Some kind of Household/Caregiving activities (such as Preparing meals, Dressing, feeding children while sitting,)  [ 1] Yes [ 2] No (if you did not, let’s skip to Q129.) | | | | | | |
| 121 | IF Yes at which trimester did you Do?  [ 1] from 1st to 3^rd^ month of pregnancy [ 2] from 4th to 6th month of pregnancy  [ 3] from 7th to 9th month of pregnancy [4] Other (describe)______________________ | | | | | | |
|  | Which of the following activities did you do? | | | **Days per week?** | | **Average time each day** | |
|  | **Light effort Household/Caregiving activities**  [ 1 ] Yes [ 2 ] No | | |  |  |  |  |
| 122 | Cleaning, general [ 1 ] Yes [ 2 ] No | | | **[** Days**]** | | **[** Hour ፡ Min **]** | |
| 123 | General infant or child care [ 1 ] Yes [ 2 ] No | | | **[** Days**]** | | **[** Hour ፡ Min **]** | |
| 124 | Disabled adult and elder care, [ 1 ] Yes [ 2 ] No | | | **[** Days**]** | | **[** Hour ፡ Min **]** | |
| 125 | Shopping (food, clothes, or other items)  [ 1 ] YES [ 2 ] NO | | | **[** Days**]** | | **[** Hour ፡ Min **]** | |
|  | **Moderate effort Household/Caregiving activities** [ 1 ] Yes [ 2 ] No | | | **Days per week?** | | **Average time each day** | |
| 126 | Kitchen activity, general, (e.g., cooking, washing dishes, cleaning up), [ 1 ] Yes [ 2 ] No | | | **[** Days**]** | | **[** Hour ፡ Min **]** | |
| 127 | Washing clothes by hand, moderate effort  [ 1 ] Yes [ 2 ] No | | | **[** Days**]** | | **[** Hour ፡ Min **]** | |
| 128 | Making bed, changing linens [ 1 ] Yes [ 2 ] No | | | **[** Days**]** | | **[** Hour ፡ Min **]** | |
| 129 | Moving, lifting light loads [ 1 ] Yes [ 2 ] No | | | **[** Days**]** | | **[** Hour ፡ Min **]** | |
| 130 | Organizing room [ 1 ] Yes [ 2 ] No | | | **[** Days**]** | | **[** Hour ፡ Min **]** | |
| 131 | Disabled adult and elder care, [ 1 ] Yes [ 2 ] No | | | **[** Days**]** | | **[** Hour ፡ Min **]** | |
| 132 | Scrubbing floors, standing, scrubbing bathroom, bathtub, [ 1 ] Yes [ 2 ] No | | | **[** Days**]** | | **[** Hour ፡ Min **]** | |
|  | **Vigorous effort Household/Caregiving activities**  [ 1 ] Yes [ 2 ] No | | | **Days per week??** | | **Average time each day** | |
| 133 | Scrubbing floors, [ 1 ] Yes [ 2 ] No | | | **[** Days**]** | | **[** Hour ፡ Min **]** | |
| 134 | Moving household items [ 1 ] Yes [ 2 ] No | | | **[** Days**]** | | **[** Hour ፡ Min **]** | |
| **S.N** | **2. Occupational activities** | | | | | | |
| 135 | At any time during this pregnancy did you practice Some of the following kind of occupational activities such as desk job (sitting/Standing with some walking, regularly lifting heavy objects, walking quickly to work with/without carrying anything? [ 1] Yes [ 2] No (if you did not, let’s skip to question 120.) | | | | | | |
| 136 | IF Yes at which trimester did you Do?  [ 1] from 1st to 3^rd^ month of pregnancy [ 2] from 4th to 6th month of pregnancy  [ 3] from 7th to 9th month of pregnancy [4] Other (describe)______________________ | | | | | | |
|  | Which of the following activities did you do? | | | | **Days per week??** | | **Average time each day** |
|  | **Light effort Occupational activities** | | | | **[** Days**]** | | **[** Hour ፡ Min **]** |
| **137** | Sitting tasks | | | | **[** Days**]** | | **[** Hour ፡ Min **]** |
| **138** | Standing tasks (e.g. Bartending, store clerk, duplicating, librarian,) | | | | **[** Days**]** | | **[** Hour ፡ Min **]** |
|  | **Moderate effort Occupational activities** | | | | **[** Days**]** | | **[** Hour ፡ Min **]** |
| **139** | Moderate effort standing tasks, lifting items continuously, packing boxes, nursing patient care) | | | | **[** Days**]** | | **[** Hour ፡ Min **]** |
|  | **Vigorous effort Occupational activities** | | | | **[** Days**]** | | **[** Hour ፡ Min **]** |
| **140** | Carrying heavy loads (e.g., bricks, tools) | | | | **[** Days**]** | | **[** Hour ፡ Min **]** |
| **141** | Carrying moderate loads upstairs, moving boxes | | | | **[** Days**]** | | **[** Hour ፡ Min **]** |
| **142** | Moving, carrying or pushing heavy objects | | | | **[** Days**]** | | **[** Hour ፡ Min **]** |
| **143** | Walking or walk downstairs or standing, carrying objects | | | | **[** Days**]** | | **[** Hour ፡ Min **]** |
| **III. Questions about Leisure time physical activity/ Physical exercise during pregnancy** | | | | | | | |
| S.N | **3. Leisure time physical activity/ Physical exercise** | | | | | | |
| 144 | In the three months before you were pregnant did you exercise regularly (2- 3 times per week for at least 3 consecutive months)? [ 1 ] Yes [ 2 ] No | | | | | | |
| 145 | At any time during this pregnancy did you practice some kind of exercise such as walking, aerobics, stretching, or prenatal exercise class? [ 1 ] Yes [ 2 ] No (explain why?__________________________)  (if you did not exercise in the first trimester let’s skip to question 140) | | | | | | |
|  | IF your answer is No which of the following is your barrier for not exercising  [ 1 ] Lacking exercise knowledge [ 3 ] Too tired  [ 2 ] Having no exercise habits [ 4 ] Having no time [ 5 ] Fear of miscarriage | | | | | | |
| 146 | IF Yes at which trimester did you Do?  [ 1] from 1st to 3^rd^ month of pregnancy [ 2] from 4th to 6th month of pregnancy  [ 3] from 7th to 9th month of pregnancy [4] Other (describe)______________________ | | | | | | |
|  | Which of the activities listed below did you do in your first trimester? (Check one or more options) | | | | | | |
|  | **Light effort Leisure time physical activity** | | | | **Days per week??** | | **Average time each day** |
| 147 | Stretching | | | | **[** Days**]** | | **[** Hour ፡ Min **]** |
|  | **Moderate effort Leisure time physical activity** | | | | **Days per week??** | | **Average time each day** |
| 148 | Walking | | | | **[** Days**]** | | **[** Hour ፡ Min **]** |
| 149 | Moderate Weightlifting | | | | **[** Days**]** | | **[** Hour ፡ Min **]** |
| 150 | Swimming | | | | **[** Days**]** | | **[** Hour ፡ Min **]** |
|  | **Vigorous effort Leisure time physical activity** | | | | **Days per week??** | | **Average time each day** |
| 151 | Running | | | | **[** Days**]** | | **[** Hour ፡ Min **]** |
| 152 | jogging | | | | **[** Days**]** | | **[** Hour ፡ Min **]** |
| 153 | General Bicycling, | | | |  | |  |
|  | **Light effort sedentary activities** | | | |  | |  |
| 154 | Lying quietly and watching television | | | | **[** Days**]** | | **[** Hour ፡ Min **]** |
|  | Sitting quietly, lying in bed awake | | | | **[** Days**]** | | **[** Hour ፡ Min **]** |
| 155 | During prenatal visits, did you receive advice to stop exercising? [ 1 ] Yes [ 2 ] No | | | | | | |
| 156 | During prenatal visits, where you directed to slow down or decrease the intensity of your  Exercising? [1] Yes [2] No | | | | | | |
